# Supplementary material for: Resolving the full spectrum of human genome variation using Linked-Reads
Source: Genome Res. 2019 Apr;29(4):635–45. doi: 10.1101/gr.234443.118 (PMC6442396; doi:10.1101/gr.234443.118)
Supplement: Supplemental Material [file supp_gr.234443.118_Supplemental_longranger-2.2.zip › longranger-2.2.tar.bz2!/lr2.2-public/lib/stan/cmdstan/stan/doc/api/html/structstan_1_1math_1_1promote__scalar__struct.html]

Stan: stan::math::promote\_scalar\_struct< T, S > Struct Template Reference


|  |  |
| --- | --- |
|  | Stan  2.5.0  probability, sampling & optimization |


- Main Page
- Related Pages
- Namespaces
- Classes
- Files

- Class List
- Class Index
- Class Hierarchy
- Class Members

All Classes Namespaces Files Functions Variables Typedefs Enumerations Enumerator Friends Macros Pages

- stan
- math
- promote\_scalar\_struct

Static Public Member Functions |
List of all members

stan::math::promote\_scalar\_struct< T, S > Struct Template Reference

General struct to hold static function for promoting underlying scalar types.
More...

`#include <promote_scalar.hpp>`

|  |  |
| --- | --- |
| Static Public Member Functions | |
| static T | apply (S x) |
|  | Return the value of the input argument promoted to the type specified by the template parameter. More... |
|  | |

## Detailed Description

### template<typename T, typename S> struct stan::math::promote\_scalar\_struct< T, S >

General struct to hold static function for promoting underlying scalar types.

Template Parameters
:   |  |  |
    | --- | --- |
    | T | return type of nested static function. |
    | S | input type for nested static function, whose underlying scalar type must be assignable to T. |

Definition at line 21 of file promote\_scalar.hpp.

## Member Function Documentation

template<typename T , typename S >

|  |  |  |  |  |  |
| --- | --- | --- | --- | --- | --- |
| static T stan::math::promote\_scalar\_struct< T, S >::apply | ( | S | *x* | ) |  |

inlinestatic

Return the value of the input argument promoted to the type specified by the template parameter.

This is the base case for mismatching template parameter types in which the underlying scalar type of template parameter `S` is assignable to type `T`.

Parameters
:   |  |  |
    | --- | --- |
    | x | input of type S. |

Returns
:   input promoted to have scalars of type T.

Definition at line 34 of file promote\_scalar.hpp.

---

The documentation for this struct was generated from the following file:

- src/stan/math/functions/promote\_scalar.hpp


---

[ Stan Home Page ]

*© 2011–2014,
Stan Development Team.*
